# Debridement, Antibiotics and Implant Retention in the Management of Periprosthetic Joint Infection: One-Year Outcomes, Epidemiology and Predictors of Failure

**DOI:** 10.3390/jcm15103728

**Published:** 2026-05-12

**Authors:** Caterina Rocchi, Alberto Bulgarelli, Vincenzo Di Matteo, Katia Chiappetta, Wim H. C. Rijnen, Guido Grappiolo, Mattia Loppini

**Affiliations:** 1 Humanitas University, Pieve Emanuele, 20090 Milan, Italy; caterina.rocchi@st.hunimed.eu; 2 Department of Biomedical Sciences, Humanitas University, Pieve Emanuele, 20090 Milan, Italy; alberto.bulgarelli@humanitas.it (A.B.); drvincenzodimatteo@gmail.com (V.D.M.); 3 IRCCS Humanitas Research Hospital, Rozzano, 20089 Milan, Italy; katia.chiappetta@humanitas.it (K.C.); guido.grappiolo@me.com (G.G.); 4 Department of Orthopedics, Radboud University Medical Center, 6525 GA Nijmegen, The Netherlands; wim.rijnen@radboudumc.nl

**Keywords:** total hip arthroplasty, total knee arthroplasty, DAIR, periprosthetic joint infection

## Abstract

**Background**: Debridement, antibiotics, and implant retention (DAIR) is the treatment of choice for early and acute hematogenous periprosthetic joint infections (PJIs), but its success rates vary widely. The primary outcome of this study was to assess 1-year implant survival and treatment failure among DAIR patients. The secondary aims were to identify the causative pathogens and potential predictors of failure. **Methods**: Patients treated with DAIR for PJI in a single center between 2017 and 2025 were included. Implant survival was evaluated using Kaplan–Meier analysis, and univariate analysis was performed to explore potential associations between baseline variables and treatment failure. **Results**: 57 patients (58 hips/knees) were included. The mean age at surgery was 67.5 ± 11.5 years, and most procedures involved the hip (93.1%, *n* = 54). During follow-up, 7 patients (12.06%) experienced treatment failure. Kaplan–Meier analysis demonstrated a 1-year implant survival of 87.9%, with most failures occurring in the early postoperative months. The microbiological profile was dominated by *Staphylococci* (51.7%, *n* = 14) and polymicrobial (24.1%, *n* = 14) species, while Gram-negative bacteria and *Enterococci* were less frequently identified. Culture-negative infections were observed in 17.2% of cases (*n* = 10). Univariate analysis identified preoperative glucose levels and BMI as significantly different between groups, although these findings should be interpreted with caution. **Conclusions**: DAIR achieved favorable short-term outcomes in this cohort. Failures occurred mainly early after treatment, and the microbiological spectrum was consistent with the typical epidemiology of PJI. BMI and perioperative glucose levels may represent potentially modifiable factors associated with treatment failure.

## 1. Introduction

Periprosthetic joint infections (PJIs) are a devastating complication of total joint arthroplasty (TJA) [1,2]. The incidence of early PJIs (within 90 days after TJA) is approximately 0.6–0.8% [3,4,5]. Early infections are most commonly caused by *Staphylococcus aureus*, coagulase-negative *Staphylococci*, and *Enterococcus* species, although Gram-negative organisms account for a relevant proportion of cases. Polymicrobial infections are also more frequent in the early postoperative setting. Compared with delayed or late infections, early PJIs are often characterized by higher virulence and a greater risk of recurrence [6,7].

Debridement, antibiotics, and implant retention (DAIR) is the preferred surgical strategy for acute postoperative and hematogenous PJIs when the implant is stable and adequate soft tissues are present. The procedure combines systemic antibiotic therapy with aggressive surgical debridement, copious irrigation of the operative field with saline and iodopovidone, and exchange of all removable modular components. The 2025 International Consensus Meeting (ICM) guidelines recommend a minimum 12-week course of targeted antimicrobial therapy following DAIR for optimal infection control. However, reported outcomes of DAIR remain heterogeneous. Recurrence rates after treatment of early PJI are 10–15% within 5 years, and about 15–50% of recurrences are attributed to the original causative organism [6,8].

Outcomes following DAIR for early PJI are variable: success rates range from 45% to 88%, with better results in cases treated within 30 days and with multidrug-sensitive organisms. DAIR failure rates are significantly higher in infections caused by methicillin-resistant *S. aureus* (18.2) and multidrug-resistant Gram-negative bacteria (55.6%) compared to multidrug-sensitive bacteria (8.3%). Importantly, recent evidence suggests that the majority of DAIR failures occur early, with approximately 70% of failures reported within the first year following the procedure [9]. Prognostic factors for DAIR failure include antimicrobial resistance, elevated preoperative ESR (>107.5), diabetes mellitus, cardiovascular disease, immunosuppression, delayed intervention, and repeat DAIR procedures [10].

Given these considerations, careful patient selection and identification of prognostic factors are essential to optimize outcomes. The primary outcome of this study was to assess 1-year implant survival and treatment failure among DAIR patients. The secondary aims were to identify the causative pathogens and clinical and microbiological variables associated with treatment failure.

## 2. Materials and Methods

### 2.1. Study Design and Patient Selection

A single-center retrospective and prospective observational study was conducted using medical records from a registry of orthopaedic surgical procedures performed at a high-volume referral center. Patients who underwent DAIR for acute PJI of the hip or knee between 2017 and 2025 were identified employing the ICD-9-CM code 996.66 (Infection and inflammatory reaction due to internal joint prosthesis). Subsequently, a second analysis was conducted, researching the words “DAIR” and/or “debridement” within surgical records. Shoulder cases were excluded from the analysis.

The study included both retrospectively and prospectively collected data. Following Ethics Committee approval on 10 January 2018, all consecutive patients were prospectively entered into the institutional arthroplasty registry. Patients treated between 2017 and 10 January 2018 were retrospectively identified through review of medical records. As all patients were included in the institutional registry, all eligible cases were included in the final analysis.

**Figure 1:**
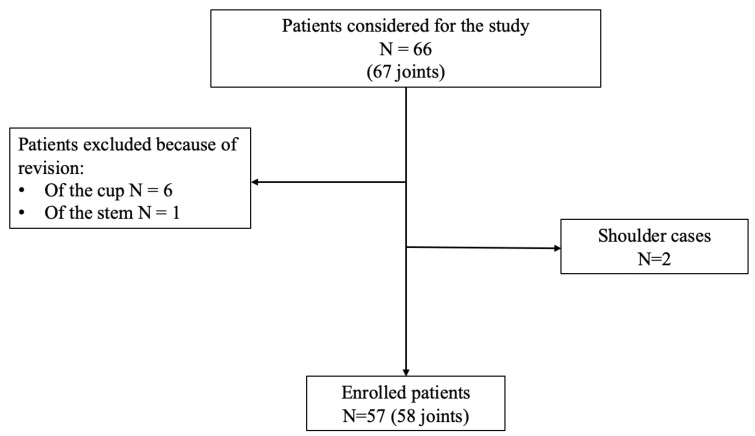
Patients’ inclusion flowchart.

Inclusion criteria comprised adult patients (≥18 years old) diagnosed with PJI who underwent DAIR for early and acute hematogenous PJI following total hip arthroplasty (THA) or total knee arthroplasty (TKA), with complete clinical and microbiological data. Patients were considered eligible for the procedure if the prosthetic components were radiographically stable. Exclusion criteria included chronic infections treated with one- or two-stage revision, incomplete anamnestic data, underage patients, and intraoperative acetabular component or stem replacement. The diagnosis of PJI was established according to the International Consensus Meeting (ICM) 2013 criteria [11]. Early postoperative PJI was defined as infection occurring within 90 days after the index arthroplasty [3]. Acute hematogenous PJI was defined as an infection in a previously well-functioning prosthetic joint, characterized by sudden-onset symptoms and presumed hematogenous spread from a distant infectious source. Cases presenting more than 90 days after the index procedure were classified as acute hematogenous PJIs if they had an abrupt clinical presentation and no prior signs of implant-related infection. Treatment failure was defined according to Delphi-based international consensus criteria. The patients’ inclusion flowchart is shown in Figure 1.

### 2.2. Surgical Technique and Microbiological Samples Analysis

All DAIR procedures were performed by experienced orthopaedic surgeons at a single high-volume referral center. A posterolateral approach was adopted for the hip and a medial parapatellar approach for the knee. The surgical procedure consisted of extensive debridement of infected and necrotic tissue, complete synovectomy, and copious pulsatile lavage with saline and iodopovidone. Whenever technically feasible, all modular components, including polyethylene liners and femoral heads, were exchanged. Fixed components were retained only if deemed stable during intraoperative assessment. Postoperatively, empiric antibiotic therapy with piperacillin–tazobactam and daptomycin was administered until microbiological culture results became available. The antibiotic regimen was subsequently de-escalated according to the identified pathogen and continued for a total of 12 weeks.

During each procedure, multiple intraoperative samples (5–7 per patient) were collected from periprosthetic tissues and synovial fluid, with each specimen placed in a separate sterile container to minimize cross-contamination. Periprosthetic tissue cultures, synovial fluid cultures, and sonication fluid cultures were performed routinely. When modular components were removed, they were sonicated in Ringer’s solution (50 Hz for 5 min) to disrupt the biofilm; the resulting fluid was then sent for microbiological analysis. All specimens underwent aerobic and anaerobic cultures, extended up to 14 days, and fungal cultures when clinically indicated. The leukocyte count of synovial fluid was determined by manual microscopy after gentle mixing to prevent sedimentation; values below 500 cells/microliter were considered negative.

### 2.3. Postoperative Management and Rehabilitation

A standardized postoperative protocol was implemented following DAIR. Early mobilization was encouraged starting on the first postoperative day, with active and passive range-of-motion (ROM) exercises tailored to patient tolerance and joint stability.

Weight-bearing was allowed as tolerated in most cases, depending on intraoperative findings and the patient’s overall condition. Physiotherapy focused on restoring ROM, preventing stiffness, and strengthening periarticular musculature. For knee procedures, strengthening exercises targeted the quadriceps, hamstrings, and calf muscles; for hip procedures, emphasis was placed on strengthening the abductors and iliopsoas.

Patients were regularly monitored through clinical assessment and laboratory evaluation, including serial C-reactive protein (CRP) measurements, to assess infection control.

### 2.4. Outcome Measures

The primary outcome was evaluating 1-year implant survival and treatment failure following DAIR. Treatment failure was defined according to Delphi consensus criteria and included recurrence of infection requiring further surgical intervention (repeat DAIR, one- or two-stage revision), chronic suppressive antibiotic therapy, or infection-related death.

Implant survival was calculated from the date of the DAIR procedure to the date of failure or last follow-up. Therefore, the one-year observation period included the postoperative antibiotic treatment phase.

Both patient and procedure-related variables were evaluated as potential predictors of failure. Microbiological variables were also analyzed, including pathogen type, the presence of multiresistant organisms, and polymicrobial infections.

### 2.5. Statistical Analysis

Due to the small sample size, only univariate analysis was performed to identify potential predictors of DAIR failure. Variables included in the analysis were age, body mass index (BMI), presence of a polymicrobial or multiresistant infection and preoperative hematologic markers. A *p*-value < 0.05 was considered statistically significant in the univariate analyses. Categorical variables were described as absolute numbers and percentages. Continuous variables were expressed as mean ± standard deviation or median with range, depending on data distribution. Categorical variables were analyzed using Pearson’s chi-square or Fisher’s exact test, while continuous variables were evaluated using the 2-sample *t*-test or Wilcoxon rank-sum test as appropriate. Reintervention-free implant survival was calculated from the date of DAIR to the date of reintervention using the Kaplan–Meier method and reported as a percentage with 95% CI during the first year of follow-up.

All statistical analyses were performed using Stata Statistical Software, Release 17 (StataCorp LLC, College Station, TX, USA). Graphs were generated using GraphPad Prism 10 (GraphPad Software, Boston, MA, USA).

## 3. Results

A total of 57 patients (58 hips/knees) were considered eligible for the study, including 54 hips (93.1%) and 4 knees (6.9%). 39 patients (68.4%) were men, and the average age at operation was 67.5 ± 11.5 years. Most of the included DAIR cases (48.3%, *n* = 28) were performed within the first month after primary surgery. Demographic characteristics are summarized in Table 1.

Microbiological cultures most frequently identified *Staphylococcus* species, which accounted for more than half of all isolates. *Staphylococcus aureus* was detected in 14 cases (24.1%), while coagulase-negative *Staphylococci* were identified in 16 cases (27.6%). *Enterococcus* species were isolated in 8 cases (13.8%), and Gram-negative organisms, including *Pseudomonas aeruginosa*, *Enterobacter*, and *Klebsiella* species, were detected in 8 cases (13.8%). Culture-negative infections occurred in 10 cases (17.2%), and polymicrobial in 14 (24.1%). Table 2 shows microbiological findings from intraoperative cultures.

**Figure 2:**
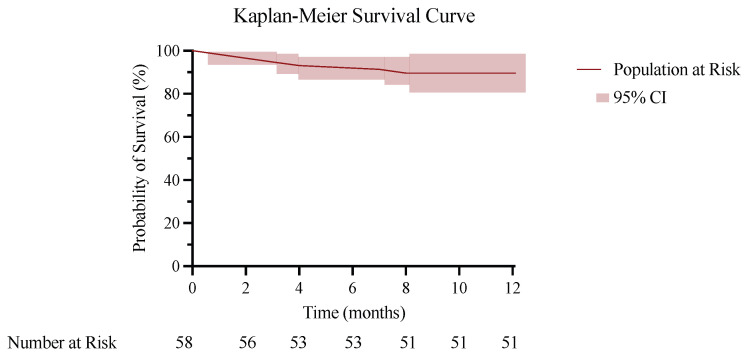
Kaplan–Meier survival curve showing freedom from reintervention following DAIR. The shaded area represents the 95% confidence interval.

All included patients were followed for one year. According to the Kaplan–Meier analysis, implant survival remained high throughout the follow-up period. At 1 month, estimated implant survival was 98.3%, decreasing slightly to 94.8% at 3 months. A further small decline was observed during the subsequent months, reaching 87.9% at 8 months. After this time point, no additional failures occurred, and implant survival remained unchanged until the end of follow-up. At 12 months, the estimated implant survival was 87.9% (95% CI 82.0–97.4%). Most failures occurred within the first 3 to 8 months following the procedure, with no additional failures observed beyond this period. Overall, seven patients experienced treatment failure. Six required reintervention during follow-up, whereas one was managed with long-term suppressive antibiotic therapy without revision surgery. Of the six patients who underwent surgery, one (16.7%) underwent spacer implantation, four (66.7%) underwent one-stage revision, and one (16.7%) underwent revision due to recurrent implant dislocation. One patient required long-term antibiotic therapy due to persistent infection. Detailed implant survival and overall treatment failure estimates derived from the Kaplan–Meier analysis are reported in Figure 2.

Univariate analysis identified a significant difference in preoperative glucose levels and BMI between the failure and non-failure groups (*p* = 0.022; *p* = 0.037), whereas no other variables were significantly associated with failure. Table 3 shows failure predictors.

## 4. Discussion

The present study evaluated the outcomes of DAIR in the treatment of PJI in a single-center cohort. The main finding is that DAIR achieved favorable short-term outcomes, with a 1-year implant survival rate of 87.9%. Most failures occurred within the first months following the procedure, after which the implant survival curve remained stable. This finding is consistent with recent literature reporting that approximately 70% of DAIR failures occur within the first year after surgery [9]. This pattern suggests that treatment failure typically manifests early after intervention, likely due to persistent biofilm-related infection or incomplete eradication of the causative organism. In contrast, patients who successfully pass this initial critical period appear to have a substantially lower risk of subsequent failure. However, due to the limited number of failure events within our cohort, it was not possible to perform a reliable subgroup analysis comparing early versus late failures in terms of clinical or microbiological characteristics.

The microbiological profile of this cohort was dominated by *Staphylococcus* species, particularly coagulase-negative *Staphylococci*, and polymicrobial infections. This finding is consistent with the well-established epidemiology of PJIs, in which *Staphylococcus aureus* and coagulase-negative *Staphylococci* are the most common pathogens [12,13]. Gram-negative bacteria and *Enterococcus* species were less frequent, but still observed in a subset of cases.

Polymicrobial infections accounted for 24.1% PJIs (*n* = 14). The incidence of polymicrobial PJIs is increasing, with reported rates ranging from 10% to 28% in large cohorts [14]. The presence of a sinus tract, prior joint revisions, and soft tissue compromise are recognized risk factors for polymicrobial infection in early PJI [15]. Compared to monomicrobial infections, they are associated with higher rates of treatment failure, a greater need for salvage procedures (such as amputation or arthrodesis), and increased morbidity. For these reasons, they require prompt recognition and appropriately targeted antibiotic treatment [16].

A proportion of infections (17.2%, *n* = 10) were culture-negative. This finding is likely influenced by prior antibiotic exposure, which may have reduced culture viability, or limitations in microbiological detection [6].

In the present analysis, age, inflammatory markers, and leukocyte counts were comparable between patients with successful outcomes and those with treatment failure. Conversely, glucose levels and BMI showed statistically significant differences; however, given the limited number of failure events, these findings should be interpreted with caution.

Perioperative hyperglycemia has been associated with an increased risk of PJI, even in non-diabetic patients [17]. Elevated preoperative blood glucose levels (e.g., ≥124 mg/dL or ≥6.9 mmol/L) and postoperative glycemia above 137 mg/dL have been linked to infection (*p* = 0.028) [18]. Although both random blood glucose and HbA1c are associated with increased PJI risk, perioperative glucose control appears to be more relevant than chronic glycemic markers alone [19,20].

Similarly, higher BMI has been shown to be linearly associated with an increased risk of PJI. Morbid obesity (BMI ≥ 40 kg/m^2^) confers a threefold or greater risk, with risk increasing progressively above a BMI of 31 kg/m^2^ [21]. Meta-analyses and large cohort studies confirm that both obesity and morbid obesity are significant, independent risk factors for PJI, with a cumulative effect when combined with other comorbidities such as diabetes [22,23]. Given that weight control represents an important modifiable factor, patients should be appropriately educated before surgery.

The identification of BMI and perioperative glucose as modifiable risk factors supports targeted preoperative optimization strategies, including metabolic control, nutritional counseling, and weight management, as well as strict perioperative glycemic monitoring, to reduce postoperative complications and improve patient outcomes [24,25].

Several factors have been proposed to influence the success of DAIR, including host characteristics, pathogen virulence, and the timing of surgical intervention. In our cohort, 48.3% of procedures were performed within 30 days after the previous surgery, 37.9% between 30 and 90 days, and 13.8% after more than 90 days. These latter cases were classified as acute hematogenous PJIs, and the procedure was performed within a short interval from symptom onset. In this context, previous studies have shown that DAIR may remain effective when performed within a few weeks after infection onset, even if the primary arthroplasty was performed long before the infection [9,26].

This study has several limitations. The retrospective part of the design may introduce recall bias, and the relatively small sample size limits the analysis’s statistical power. In particular, the low number of failure events precluded multivariable modeling to identify predictors of failure. Additionally, microbiological heterogeneity and variations in patient comorbidities may have influenced treatment success. Another limitation is the predominance of hip cases, with only a small proportion of knee arthroplasties included. Although DAIR principles are similar between joints, this imbalance may limit the generalizability of our findings. Despite these limitations, the present study supports the role of DAIR as an effective treatment option in selected cases of PJI. Further studies with larger cohorts are warranted to better define the factors influencing treatment success and to refine patient selection for this procedure.

## 5. Conclusions

DAIR demonstrated favorable short-term outcomes in this cohort, with high implant survival and overall treatment success at one year. The microbiological profile was predominantly characterized by *Staphylococcus* species and polymicrobial infections, reflecting the typical epidemiology of PJI. No demographic, clinical, or hematologic variables were significantly associated with treatment failure. However, preoperative glucose levels and BMI differed between groups in the univariate analysis and may represent potentially modifiable factors. Further studies with larger cohorts are warranted to better define the determinants of treatment success and to refine patient selection for this procedure.

## Figures and Tables

**Table 1:** Demographic and clinical characteristics of the study population.

Variable	Value
Number of patients	57
Age (years)	67.5 ± 11.5
Body mass index (kg/m^2^)	28.2 ± 5.1
Male sex	39 (68.4%)
Female sex	18 (31.6%)
Hip joints	54 (93.1%)
Knee joints	4 (6.9%)
Active smokers	8 (14.0%)
ASA I	17 (29.8%)
ASA II	29 (50.9%)
ASA III	11 (19.3%)
ASA IV	0 (0%)
Time from Previous Surgery < 30 days	28 (48.3%)
Time from Previous Surgery 30–90 days	22 (37.9%)
Time from Previous Surgery > 90 days	8 (13.8%)

**Table 2:** Microbiological findings from intraoperative cultures.

Pathogen	*n* (%)
*Staphylococcus aureus* (MSSA/MRSA)	14 (24.1%)
Coagulase-negative *Staphylococcus* spp.	16 (27.6%)
*Enterococcus* spp.	8 (13.8%)
Gram-negative bacteria (*Pseudomonas*, *Enterobacter*, *Klebsiella*)	8 (13.8%)
*Cutibacterium acnes*	2 (3.4%)
*Streptococcus pyogenes*	1 (1.7%)
*Corynebacterium* spp.	1 (1.7%)
*Bacillus pumilus*	2 (3.4%)
Culture-negative infections	10 (17.2%)
Polymicrobial	14 (24.1%)

**Table 3:** Univariate analysis of potential risk factors for treatment failure. Continuous variables are expressed as mean ± standard deviation, and categorical variables as number (percentage). MCV: mean corpuscular volume.

Variable	Failure (*n* = 7)	No Failure (*n* = 51)	*p*-Value
Age (years)	65.4 ± 9.4	68.2 ± 12.2	0.504
Body mass index (kg/m^2^)	30.7 ± 0.8	28.2 ± 5.0	0.037
Polymicrobial infection, *n* (%)	1 (14.3%)	13 (25.5%)	1.000
Multiresistant organism, *n* (%)	1 (14.3%)	14 (28.6%)	0.661
Hemoglobin (g/dL)	11.9 ± 0.9	11.8 ± 2.3	0.760
Hematocrit (%)	36.2 ± 2.3	35.4 ± 7.5	0.554
Platelets ($10^{3}$/µL)	333 ± 100.6	334 ± 111.3	0.982
Leukocytes ($10^{3}$/µL)	9.21 ± 2.56	8.79 ± 3.34	0.727
C-reactive protein	13.7 ± 11.1	16.1 ± 31.1	0.720
Creatinine	0.73 ± 0.16	0.86 ± 0.22	0.156
Ferritin	228.6 ± 241.6	132.0 ± 112.2	0.425
Glucose	91.7 ± 11.4	106.6 ± 18.2	0.022
Neutrophils	6.92 ± 2.77	6.02 ± 2.94	0.480
Lymphocytes	1.52 ± 0.50	1.59 ± 0.64	0.751
Monocytes	0.60 ± 0.18	0.68 ± 0.30	0.376
Eosinophils	0.23 ± 0.19	0.22 ± 0.20	0.907
Basophils	0.03 ± 0.05	0.02 ± 0.04	0.611
Erythrocytes	4.11 ± 0.31	4.10 ± 0.67	0.946
MCV	88.5 ± 6.6	87.6 ± 13.2	0.793

## Data Availability

Data supporting reported results can be found in a repository (Zenodo).
